# Regulation of Enterocyte Brush Border Membrane Primary Na-Absorptive Transporters in Human Intestinal Organoid-Derived Monolayers

**DOI:** 10.3390/cells13191623

**Published:** 2024-09-28

**Authors:** Jennifer Haynes, Balasubramanian Palaniappan, John M. Crutchley, Uma Sundaram

**Affiliations:** Department of Clinical and Translational Sciences, Joan C. Edwards School of Medicine, Marshall University, 1600 Medical Center Drive, Huntington, WV 25701, USA

**Keywords:** Na absorption, human enterocytes, intestinal organoids, jejunal monolayers, siRNA, NHE3, SGLT1

## Abstract

In the small intestine, sodium (Na) absorption occurs primarily via two apical transporters, Na-hydrogen exchanger 3 (NHE3) and Na-glucose cotransporter 1 (SGLT1). The two primary Na-absorptive pathways were previously shown to compensatorily regulate each other in rabbit and rat intestinal epithelial cells. However, whether NHE3 and SGLT1 regulate one another in normal human enterocytes is unknown, mainly due to a lack of appropriate experimental models. To investigate this, we generated 2D enterocyte monolayers from human jejunal 3D organoids and used small interfering RNAs (siRNAs) to knock down NHE3 or SGLT1. Molecular and uptake studies were performed to determine the effects on NHE3 and SGLT1 expression and activity. Knockdown of NHE3 by siRNA in enterocyte monolayers was verified by qPCR and Western blot analysis and resulted in reduced NHE3 activity. However, in NHE3 siRNA-transfected cells, SGLT1 activity was significantly increased. siRNA knockdown of SGLT1 was confirmed by qPCR and Western blot analysis and resulted in reduced SGLT1 activity. However, in SGLT1 siRNA-transfected cells, NHE3 activity was significantly increased. These results demonstrate for the first time the functionality of siRNA in patient-derived organoid monolayers. Furthermore, they show that the two primary Na absorptive pathways in human enterocytes reciprocally regulate one another.

## 1. Introduction

The primary function of the mammalian small intestine, which consists of three distinct segments—the duodenum, jejunum, and ileum—is to absorb nutrients, electrolytes, and water for use throughout the body. The intestinal lining is composed of a highly folded epithelium that is organized into crypt regions and tiny finger-like projections called villi, which help to increase the surface area of the small intestine [1]. In addition, the apical membrane of villus cells is folded into protrusions called microvilli to further increase the intestinal surface area and thereby facilitate absorptive processes. While the intestinal stem and progenitor cells are located in the crypts, most differentiated intestinal epithelial cell types are located in the villi [2]. This includes enterocytes, which transport nutrients, electrolytes, and water across the intestinal barrier, and account for ~80% of all intestinal epithelial cells [1]. Gastrointestinal (GI) absorption of nutrients and electrolytes is physiologically regulated by various factors including hormones and other signaling molecules. Intestinal absorption is known to be dysregulated in GI disorders, causing malabsorption [3,4], and in inflammatory bowel diseases (e.g., Crohn’s disease, ulcerative colitis) [5,6,7]; however, the underlying molecular mechanisms are not well understood. In addition, there is emerging evidence in obesity that enhanced intestinal absorption of sodium (Na) and glucose is likely critical in the pathogenesis of hypertension and diabetes, respectively [8]. Thus, a greater understanding of how intestinal absorption is regulated would allow for the development of better treatments for GI and other diseases.

Long-term patient-derived organoid cultures are a valuable in vitro model for studying human intestinal physiology and regulation. Intestinal epithelial organoids are generated from intestinal stem cells isolated from adult tissue and can both self-renew and differentiate in culture [1]. While intestinal organoids grow within an extracellular matrix as 3D structures containing both “crypt-like” and “villus-like” domains [9], the apical membrane faces the inside of the organoid, and is therefore not easily accessible. To facilitate experiments in which the apical domain is directly exposed to the medium, 2D organoid-derived monolayer culture models have been established [10,11]. Human intestinal organoid-derived monolayers have been successfully used to study host–pathogen interactions [10,11,12], epithelial barrier function [13], and various transport processes, including secretion of hormones, growth factors, and ions [14,15,16], and absorption of glucose [17]. Moreover, recent work from our laboratory demonstrated that human jejunal organoid monolayers can be used as an in vitro model to investigate the regulation of nutrient and electrolyte absorption [18]. In the absence of suitable normal human cell lines, the development of intestinal organoid monolayers has been a major breakthrough for studying human intestinal epithelial transport physiology and other fundamental cellular processes in vitro.

Absorption of nutrients and electrolytes is mediated by different transporter proteins that are localized to the apical or brush border membrane (BBM) of enterocytes [19]. The two main pathways of Na absorption in the human small intestine are BBM transporters Na-hydrogen exchanger 3 (NHE3; SLC9A3) and Na-glucose cotransporter 1 (SGLT1; SLC5A1). NHE3 exchanges one luminal Na ion for one intracellular H ion and is the principal NHE isoform expressed in the small intestinal BBM [20]. SGLT1 is a secondary active transporter that translocates two Na ions coupled with one glucose molecule into cells and is also the most abundant glucose transporter in the small intestinal BBM [21]. The favorable intracellular Na gradient for NHE3 and SGLT1 in enterocytes is provided by the sodium–potassium adenosine triphosphatase (Na-K-ATPase) pump at the basolateral membrane.

There is emerging evidence that the two primary Na absorptive pathways can regulate one another [22], which may be mediated by nitric oxide (NO) signaling in some contexts [23,24,25]. NO is an essential biological messenger molecule that participates in multiple GI and hepatic physiological and pathological processes. Low levels of NO (constitutive nitric oxide; cNO) regulate various normal intestinal physiological functions including GI motility, mucosal permeability, and blood flow regulation [26]. Consistent with our previous studies performed in mammalian intestinal epithelial cell models [25], we recently showed that enhanced intracellular cNO inhibits NHE3 but stimulates SGLT1 in differentiated human jejunal organoid-derived monolayers [18]. In a separate study, NHE3 and SGLT1 were shown to reciprocally regulate one another in vitro in rat intestinal epithelial cells [27]. However, it is not known if NHE3 and SGLT1 regulate one another in human enterocytes, mainly due to a lack of appropriate experimental models.

Therefore, the aim of this study was to evaluate the use of small interfering RNA (siRNA) technology to determine if NHE3 and SGLT1 regulate one another in normal human enterocytes. To this end, we performed siRNA-mediated knockdown of the two primary apical Na transporters in human jejunal organoid-derived monolayers, followed by molecular and uptake studies to determine the effects on NHE3 and SGLT1 expression and activity.

## 2. Materials and Methods

### 2.1. Antibody Generation

Polyclonal antibodies against SGLT1 were raised in rabbit using Invitrogen (Waltham, MA, USA) custom antibody services. A peptide corresponding to amino acids 581–599 of human SGLT1 (QEGPKETIEIETQVPEKKK) was used as the immunogen [28] and for antibody affinity purification.

### 2.2. Intestinal Organoid Culture

Previously established jejunal organoids were cultured in 3D as described (organoid model MU-007) [18]. Organoids were generated from jejunal biopsy tissue obtained during upper endoscopy from a healthy donor without clinical evidence of small intestinal pathophysiology. Histologic evaluation of adjacent intestinal biopsy tissue confirmed that the jejunum was normal. This study was approved by the Institutional Review Board of Marshall University (protocol 964144).

Organoid growth medium was composed of Advanced DMEM/F-12 (ADF; Gibco, Waltham, MA, USA), 2 mM GlutaMAX (Gibco), 10 mM HEPES (Gibco), 100 U/mL Pen-Strep (Gibco), 40% Wnt-3a conditioned medium, 10% Rspo1 conditioned medium, 1X B-27 Supplement (Gibco), 1.25 mM N-acetyl-L-cysteine (Sigma. Burlington, MA, USA), 100 ng/mL mouse Noggin (Peprotech, Cranbury, NJ, USA), 50 ng/mL mouse EGF (Gibco), 0.5 µM A83-01 (Tocris, Minneapolis, MN, USA), 10 nM Gastrin I (Sigma), and 10 µM SB202190 (Sigma). Conditioned media was prepared from L-Wnt-3a and 293T-R-Spondin1 producer cell lines as described [18]. When passaging organoids, the medium also contained 10 µM Y-27632 and 2.5 µM CHIR 99021. Organoids grew embedded in 50 μL droplets of growth-factor-reduced phenol red-free Matrigel basement membrane matrix (Corning, Corning, NY, USA) overlaid with 600 μL of growth medium per well in 24-well plates. The medium was changed every 2 or 3 days, and cultures were passaged every 7 days. Organoid cultures were maintained at 37°C in a humidified incubator at 6% CO_2_, for up to 12 passages. All cultures were confirmed to be negative for mycoplasma.

### 2.3. Organoid-Derived Monolayers

For monolayer plating, single-cell suspensions were prepared from organoid cultures as previously described [18]. Monolayers were maintained in differentiation medium (DFM), composed of ADF, 2 mM GlutaMAX, 10 mM HEPES, 100 U/mL Pen-Strep, 15% Rspo1 conditioned medium, 1X B-27 Supplement, 1.25 mM N-acetyl-L-cysteine, 100 ng/mL mouse Noggin, 50 ng/mL mouse EGF, 500 nM A83-01, and 10 nM Gastrin I. When seeding monolayers, the medium also contained 10 µM Y-27632. The medium was changed every 2 or 3 days, and experiments were conducted on day 5 or 6 using confluent monolayers. For the final media change, DFM was modified to be antioxidant-free by substituting standard B-27 Supplement with B-27 minus antioxidants (Gibco) and omitting N-acetyl-L-cysteine.

### 2.4. Cell Staining

For immunocytochemistry, monolayers were grown on Nunc Lab-Tek II CC^2^ Glass chamber slides (Thermo Scientific, Waltham, MA, USA) and processed as previously described [18]. Briefly, monolayers were washed three times with Phosphate-Buffered Saline (PBS) + Ca/Mg, fixed with PBS 4% paraformaldehyde for 12 min, permeabilized with PBS 0.5% Triton X-100 for 10 min, and then blocked with PBS 3% Bovine Serum Albumin (BSA) for 1 h. Fixed cells were incubated with rabbit anti-ZO-1 polyclonal antibody (Invitrogen, 40–2200; 1:200) for 1 h. Bound antibodies were labeled with Alexa Fluor 594-conjugated goat anti-rabbit secondary antibody (Invitrogen; 1:500) for 1 h in the dark. For mounting, ProLong Diamond Antifade (Invitrogen) was applied to the slides and coverslips were placed on top, then the samples were cured for 24 h. Fluorescence images were acquired using a 20× fluorite, LWD, 0.45NA/6.23WD air objective on an EVOS FL Auto 2 imaging system (Invitrogen).

For staining of alkaline phosphatase activity, monolayers grown on Nunc Lab-Tek Permanox Plastic chamber slides (Thermo Scientific) were washed three times with PBS + Ca/Mg, fixed with PBS 4% paraformaldehyde for 10 min, and then washed twice with Tris Buffered Saline (TBS; 25 mM Tris-HCl, 0.15 M NaCl, pH 7.4). Fixed cells were incubated with BCIP/NBT Alkaline Phosphatase Substrate working solution (Vector Laboratories, Newark, CA, USA) for 6 h in the dark. After three washes with TBS, coverslips were mounted onto slides with VectaMount AQ Mounting Medium (Vector Laboratories). Bright-field images were acquired using a 10× plan achromatic 0.25NA phase objective and a Moticam 10+ digital camera (Motic, Xiamen, China) mounted on a trinocular inverted microscope (VWR).

### 2.5. RNA Extraction

Organoids were recovered from the basement membrane matrix using Cell Recovery Solution (Corning) as previously described [18]. Organoids were then lysed in Buffer RLT (Qiagen, Germantown, MD, USA). For monolayer cultures, cells were scraped directly in Buffer RLT. All lysates were homogenized using a QIAshredder spin column (Qiagen), and total RNA was extracted using RNeasy Mini Kit (Qiagen) following the manufacturer’s protocol, including on-column DNase digestion. RNA concentration was determined using a NanoDrop 2000 Spectrophotometer (Thermo Scientific).

### 2.6. qPCR Analysis

Quantitative real-time polymerase chain reaction (qPCR) was performed using Superscript IV (Invitrogen) to synthesize first-strand cDNA from total RNA (500 ng). The cDNAs were amplified using PowerUP SYBR Green Master Mix (Applied Biosystems, Foster City, CA, USA) following the manufacturer’s protocol and detected on a StepOnePlus Real-Time PCR System (Applied Biosystems). Relative mRNA expression was calculated using the delta–delta CT method. Housekeeping genes *ACTB* and *TBP* were used for normalization. Primer sequences: *ACTB* Fwd: ACCATGGATGATGATATCGCC, *ACTB* Rev: GCCTTGCACATGCCGG [29]; *FABP2* Fwd: GCTGCAAGCTTCCTTTTCAC, *FABP2* Rev: CTGAAATCATGGCGTTTGAC [30]; *LGR5* Fwd: CCTACCTAGACCTCAGTATGAAC, *LGR5* Rev: CCCTTGGGAATGTATGTCAGA; *MKI67* Fwd: GAGGTGTGCAGAAAATCCAAA, *MKI67* Rev: CTGTCCCTATGACTTCTGGTTGT [31]; *NHE3* Fwd: GTCTTCCTCAGTGGGCTCAT, *NHE3* Rev: ATGAGGCTGCCAAACAGG [16]; *SGLT1* Fwd: GCATCGCCTGGGTGCCCATT, *SGLT1* Rev: GCACCGTGCTGCTCTAGCCC [28]; *TBP* Fwd: GGGCATTATTTGTGCACTGAGA, *TBP* Rev: TAGCAGCACGGTATGAGCAACT [32].

### 2.7. siRNA Transfection

*Silencer* Select pre-designed siRNA (Ambion, Waltham, MA, USA) targeting human *NHE3* (*SLC9A3*; s13029), human *SGLT1* (*SLC5A1*; s12953), or the non-targeting negative control siRNA (Negative Control No. 1) was used for transfection experiments. The siRNAs (final concentration 15 nM) were introduced into cell monolayers one day after plating, when cells were approximately 60% confluent. Transfections were performed using Lipofectamine RNAiMAX Reagent (Invitrogen), with minor modifications to the manufacturer’s protocol. For 24-well plates, 1 μL siRNA and 3 μL lipid reagent were added to cell monolayers in a total volume of 63 μL Opti-MEM medium (Gibco), and volumes were scaled down proportionately for 96-well plates. The monolayer medium (DFM) was changed on day 2 and day 4, and assays were conducted on day 5.

### 2.8. Protein Extraction

Whole cell extracts were prepared from monolayers as previously described [18]. The lysates were diluted to a final concentration of 1X Laemmli Sample Buffer (Bio-Rad, Hercules, CA, USA) plus β-mercaptoethanol and the samples were incubated at 70 °C for 10 min.

For the preparation of total cell membranes, monolayers were washed twice with cold PBS, scraped in ice-cold Subcellular Fractionation Buffer [20 mM HEPES-Tris pH 7.4, 250 mM Sucrose, 10 mM KCl, 2 mM MgCl_2_, 1 mM Ethylenediaminetetraacetic acid (EDTA), 1 mM Ethylene glycol-bis(β-aminoethyl ether)-N,N,N′,N′-tetraacetic acid (EGTA), 1 mM Dithiothreitol (DTT), and Protease Inhibitor Cocktail], then transferred to cold microcentrifuge tubes and incubated on ice for 15 min. The cell suspensions were passed through a 27-gauge needle 20 times, and then the tubes were rotated at 4 °C for 20 min. The cellular debris was pelleted by centrifugation at 10,000 × g for 5 min (4 °C). The membrane fraction was pelleted by ultracentrifugation of the supernatant at 37,000× *g* for 2 h (4 °C). The resulting supernatant (cytosolic fraction) was concentrated by centrifugation through a filter unit, MWCO 10 kDa (Pierce, Thermo Scientific, Waltham, MA, USA) at 15,000× *g* for 10–15 min to a volume of 75–100 μL, and protein concentration was measured as described above. The cytosolic fractions were mixed with 4X Laemmli Buffer without reducing the agent to a final concentration of 1X, and the total cell membrane pellets were resuspended in 1X Laemmli Buffer without a reducing agent, at a volume equivalent to the final volume of the cytosolic fraction. Samples were incubated at 65 °C for 15 min.

### 2.9. Western Blot Analysis

Western blots were carried out as previously described [18], using 10–20 µg of protein and 8% or 10% SDS-PAGE. Custom-made rabbit anti-SGLT1 primary antibodies were used at 1:1000 and incubated overnight at 4 °C.

### 2.10. Uptake Studies

Uptake studies to assess SGLT1 activity or NHE3 activity were performed as previously described [18].

### 2.11. Statistical Analysis

Data are shown as mean ± standard error of the mean (SEM). *p* values were derived via a two-tailed Student’s *t* test using Prism 9 software (GraphPad, San Diego, CA, USA). Differences were considered statistically significant when *p* < 0.05.

## 3. Results

### 3.1. Characterization of Human Jejunal Organoid-Derived Intestinal Epithelial Cell Monolayers

**Figure 1:**
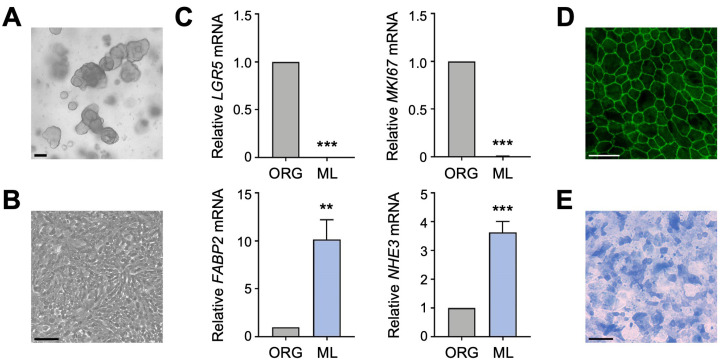
Characterization of human intestinal organoid-derived monolayers. (**A**,**B**) Phase contrast images of human jejunal organoids (**A**) and differentiated organoid-derived monolayers (**B**). Scale bars, 100 µm. (**C**) Quantitative polymerase chain reaction (qPCR) analysis showing the expression of marker genes in undifferentiated organoids (ORG) versus differentiated organoid monolayers (ML). Numbers are relative to ORGs and normalized to the geometric mean of *ACTB* and *TBP*. Data are represented as mean ± standard error of the mean (SEM) (*n* = 4 experiments). Statistical significance was determined by unpaired *t* test (**, *p* < 0.01; ***, *p* < 0.001). (**D**) Representative immunofluorescence image of tight junction marker ZO-1 in differentiated jejunal monolayers. Scale bar, 50 µm. (**E**) Representative brightfield image of alkaline phosphatase staining of monolayers. Scale bar, 100 µm.

To determine if the two primary apical Na transporters, NHE3 and SGLT1, regulate each other in human enterocytes, we utilized a human intestinal organoid-derived monolayer culture model. Previously established 3D organoids (Figure 1A), derived from histologically normal jejunal biopsy tissue, and organoid-derived 2D monolayers (Figure 1B) were cultured as described [18]. Differentiation of jejunal monolayers was verified by qPCR analysis of intestinal epithelial cell markers. Compared to undifferentiated organoids, expression of the intestinal stem cell marker *LGR5* and the proliferation marker *MKI67* was substantially decreased in differentiated monolayers (Figure 1C), in accordance with an earlier study performed in human duodenal organoid-derived monolayers [16]. This decrease was accompanied by a concomitant increase in expression of the enterocyte marker *FABP2* and BBM transporter *NHE3* (Figure 1C). These gene expression results are consistent with our previous work showing that expression of brush border proteins Villin, Ezrin, and NHE3 was increased in differentiated jejunal monolayers compared to undifferentiated organoids [18]. The integrity and apical–basal polarity of intestinal epithelial cell monolayers was demonstrated by immunostaining for the tight junction protein ZO-1 (Figure 1D) [18], and differentiation of monolayers into the enterocyte lineage was further confirmed by staining for alkaline phosphatase activity (Figure 1E). Thus, human jejunal organoid-derived monolayers are composed of differentiated enterocytes and are therefore a suitable in vitro model to investigate the regulation of apical Na transporter function in normal human enterocytes.

### 3.2. Silencing of NHE3 Expression Regulates the Functional Activity of SGLT1 in Human Jejunal Monolayers

**Figure 2:**
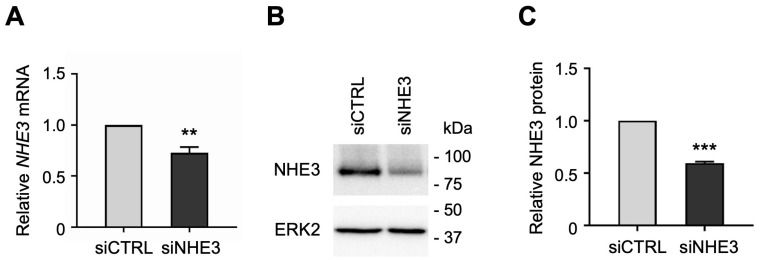
Small interfering RNA (siRNA)-mediated knockdown of sodium–hydrogen exchanger 3 (NHE3) in human jejunal organoid-derived monolayers. (**A**) qPCR analysis of *NHE3* gene expression in differentiated jejunal monolayers transfected with negative control siRNA (siCTRL) or NHE3 siRNA (siNHE3). Numbers are relative to siCTRL and normalized to *ACTB*. Data are represented as mean ± SEM (*n* = 4 experiments). (**B**) Representative Western blots showing NHE3 protein expression in whole-cell extracts of siRNA-transfected monolayers. ERK2 was used as a loading control. (**C**) Densitometric analysis of NHE3 protein expression in siRNA-transfected monolayers. Numbers are relative to siCTRL and normalized to ERK2. Data are represented as mean ± SEM (*n* = 3 experiments). Statistical significance was determined by unpaired *t* test (**, *p* < 0.01; ***, *p* < 0.001).

NHE3 and SGLT1 were previously shown to regulate one another in a nontransformed rat intestinal epithelial cell line [27]. However, these findings have not been examined in normal human intestinal epithelial cells due to lack of a suitable cell culture model. To determine whether NHE3 expression regulates SGLT1 activity in human enterocytes, we silenced NHE3 expression by transfecting jejunal monolayers with human NHE3-targeting small interfering RNA (siRNA) sequences. While siRNA technology is routinely used to induce transient gene silencing in immortalized cell lines, it has not been previously demonstrated in organoid-derived monolayer cultures. The siRNA-mediated knockdown of NHE3 was verified by qPCR analysis, which showed that *NHE3* mRNA expression was significantly reduced in differentiated NHE3 siRNA-transfected (siNHE3) monolayers compared to control cells transfected with non-targeting negative control siRNA sequences (siCTRL; Figure 2A). Silencing of NHE3 expression was further validated by Western blot analysis, which showed that NHE3 protein expression was significantly reduced in whole cell extracts from siNHE3 monolayers compared to siCTRL monolayers (Figure 2B,C and Figure S1).

**Figure 3:**
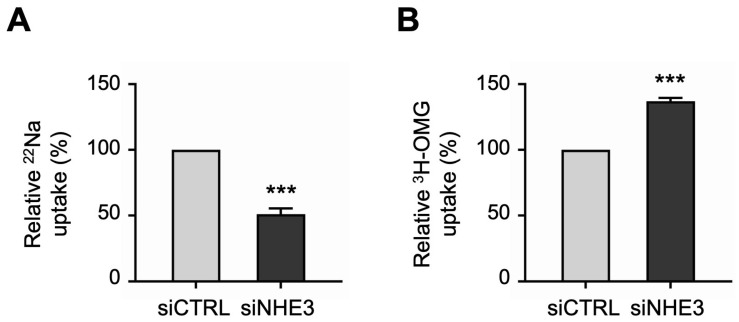
Functional activities of the two primary apical sodium (Na) transporters in NHE3 siRNA-transfected human jejunal organoid-derived monolayers. (**A**) The activity of NHE3 was measured as EIPA-sensitive uptake of ^22^Na in differentiated NHE3 siRNA-transfected (siNHE3) monolayers. Uptake values are relative to monolayers transfected with negative control siRNA (siCTRL). (**B**) The activity of SGLT1 was measured as the phlorizin-sensitive Na-dependent uptake of ^3^H-OMG in the siNHE3 monolayers. Uptake values are relative to siCTRL monolayers. Data are represented as mean ± SEM (*n* = 4 experiments, each containing triplicate reactions). Statistical significance was determined by unpaired *t* test (***, *p* < 0.001). EIPA, 5-(N-Ethyl-N-isopropyl)amiloride. OMG, 3-*O*-Methyl-D-glucopyranose.

Next, we performed uptake studies to examine the functional activities of the two primary apical Na transporters in differentiated NHE3 siRNA-transfected monolayers. NHE3 activity was measured directly as the Na-dependent, 5-(N-Ethyl-N-isopropyl)amiloride (EIPA)-sensitive uptake of the radioisotope ^22^Na. Consistent with the silencing of NHE3 expression described above, ^22^Na uptake studies demonstrated that NHE3 functional activity was significantly decreased in siNHE3 monolayers compared to siCTRL monolayers (Figure 3A). SGLT1 activity was measured directly as the Na-dependent, phlorizin-sensitive uptake of the radiolabeled glucose analogue ^3^H-3-*O*-Methyl-D-glucopyranose (^3^H-OMG). In contrast to the reduced functional activity of NHE3, ^3^H-OMG uptake studies demonstrated that SGLT1 functional activity was significantly increased in siNHE3 monolayers (Figure 3B).

### 3.3. Silencing of SGLT1 Expression Regulates the Functional Activity of NHE3 in Human Jejunal Monolayers

**Figure 4:**
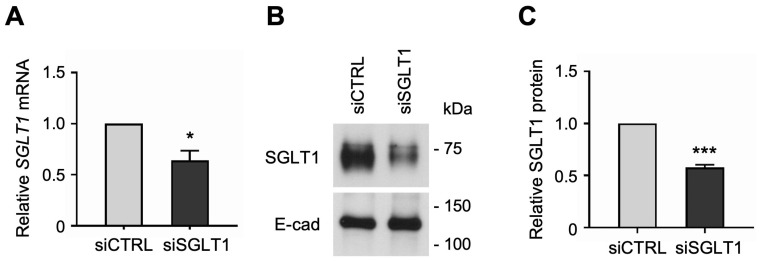
siRNA-mediated knockdown of Na-glucose cotransporter 1 (SGLT1) in human jejunal organoid-derived monolayers. (**A**) qPCR analysis of *SGLT1* gene expression in differentiated jejunal monolayers transfected with negative control siRNA (siCTRL) or SGLT1 siRNA (siSGLT1). Numbers are relative to siCTRL and normalized to *ACTB*. Data are represented as mean ± SEM (*n* = 3 experiments). (**B**) Representative Western blots showing SGLT1 protein expression in total cell membranes isolated from siRNA-transfected monolayers. The epithelial cell adhesion protein E-cadherin (E-cad) was used as a loading control. (**C**) Densitometric analysis of SGLT1 protein expression in siRNA-transfected monolayers. Numbers are relative to siCTRL and normalized to E-cad. Data are represented as mean ± SEM (*n* = 3 experiments). Statistical significance was determined by unpaired *t* test (*, *p* < 0.05; ***, *p* < 0.001).

Similarly, to determine whether SGLT1 expression regulates NHE3 activity in human enterocytes, we silenced SGLT1 expression by transfecting jejunal monolayers with human SGLT1-targeting siRNA sequences. The siRNA-mediated knockdown of SGLT1 was verified by qPCR analysis, which showed that *SGLT1* mRNA expression was significantly reduced in differentiated SGLT1 siRNA-transfected (siSGLT1) monolayers compared to siCTRL monolayers (Figure 4A). Silencing of SGLT1 expression was further validated by Western blot analysis, which showed that SGLT1 protein expression was significantly reduced in total cell membrane extracts from siSGLT1 monolayers compared to siCTRL monolayers (Figure 4B,C and Figure S2).

**Figure 5:**
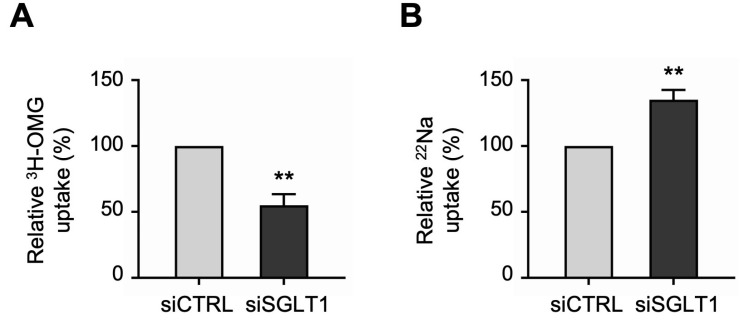
Functional activities of the two primary apical sodium transporters in SGLT1 siRNA-transfected human jejunal organoid-derived monolayers. (**A**) The activity of SGLT1 was measured as the phlorizin-sensitive Na-dependent uptake of ^3^H-OMG in differentiated SGLT1 siRNA-transfected (siSGLT1) monolayers. Uptake values are relative to monolayers transfected with negative control siRNA (siCTRL). (**B**) The activity of NHE3 was measured as EIPA-sensitive uptake of ^22^Na in siSGLT1 monolayers. Uptake values are relative to siCTRL monolayers. Data are represented as mean ± SEM (*n* = 4 experiments, each containing triplicate reactions). Statistical significance was determined by unpaired *t* test (**, *p* < 0.01).

Uptake studies showed that the functional activities of the two primary apical Na transporters were differentially regulated in SGLT1 siRNA-transfected monolayers. Consistent with the silencing of SGLT1 expression described above, ^3^H-OMG uptake studies demonstrated that SGLT1 functional activity was significantly decreased in siSGLT1 monolayers compared to siCTRL monolayers (Figure 5A). However, in contrast to the reduced functional activity of SGLT1, ^22^Na uptake studies demonstrated that NHE3 functional activity was significantly increased in siSGLT1 monolayers (Figure 5B).

Thus, through novel use of siRNA-mediated knockdown in intestinal organoid-derived monolayers, these experiments demonstrate that the two primary apical Na transporters, NHE3 and SGLT1, reciprocally regulate one another in human enterocytes. This is consistent with the compensatory regulation between NHE3 and SGLT1 observed in mammalian intestinal cell models [24,25,27], and therefore serves as proof of concept for using siRNA technology in patient-derived organoid monolayers.

## 4. Discussion

Altered intestinal absorption is a key feature of various GI disorders associated with malabsorption [3,4] and inflammatory bowel diseases [5,6,7]. Hence, a better understanding of the regulation of intestinal absorption by BBM transporters, their regulatory proteins, and associated signaling pathways would enable the advancement of more effective treatments for GI diseases. Mechanistic studies on intestinal transport have been performed using a variety of different and complementary experimental models including in vivo animal models, ex vivo human tissue, and a limited number of in vitro cell culture-based models [23,33,34,35,36,37]. Until recently, human cell culture models for studying intestinal epithelial cell transport have generally used colon cancer cell lines such as Caco-2, HT-29, and T84 [38,39], which are immortalized and contain vastly mutated genomes in addition to altered transcriptional and metabolic profiles [40]. It is therefore not clear if these models accurately reflect normal expression, function, and regulation of intestinal epithelial cell transporters, but so far, immortalized colon cancer cells lines have demonstrated limited clinical translatability [41,42]. The development and utilization of nontransformed, nonimmortalized cell culture models derived from healthy human intestinal tissue is therefore critical for in vitro investigation of intestinal physiology and pathophysiology in translational research.

The establishment of patient-derived intestinal 3D organoid culture models [9] and their increasing use over the last decade has greatly facilitated the investigation of human intestinal physiology and regulation [43]. Furthermore, the recent development of organoid-derived 2D monolayer culture models has been instrumental for studies in which access to the apical surface is important [10,11,44]. This includes research on intestinal transport processes such as secretion of growth factors, hormones, and ions [14,15,16], and absorption of Na [18] and glucose [17,18]. Along with our previous study [18], here we demonstrate that jejunal organoid monolayers are an ideal in vitro model to investigate the regulation of apical transporter function in human enterocytes.

Modifying cellular gene expression is key to understanding the function and biological role of genes. There are multiple tools for the genetic engineering of cultured cells, involving the introduction of gene-editing components by viral or non-viral means [45]. RNA interference (RNAi) is a popular method to silence or “knock down” gene expression in cells either transiently by siRNA or stably by short hairpin RNA (shRNA). More recently, gene-editing systems have been widely adapted to eliminate or “knock out” gene expression in 2D and 3D cultures, such as Clustered Regularly Interspaced Palindromic Repeats (CRISPR)/Cas9 [45]. While complete gene knockout is often preferred, there are some drawbacks of CRISPR technology including Cas9 off-target activity [46], as well as the cost and labor-intensive process of guide RNA design, viral packaging and infection, isolation of single cells, expansion of clones, and verification of correct targeting [47]. Furthermore, in many cases, experimental design may only require the transient knockdown of gene expression, for which siRNA has proven to be very successful. Some of the advantages of siRNA-mediated knockdown are that it is simple, convenient, economical, and effective in all mammalian somatic cell types [45]. Generally, siRNAs are transiently introduced into cells either by lipofection or electroporation, so time-consuming viral packaging and infection and/or subsequent selection steps are not required. While the use of siRNA for gene knockdown is common in 2D immortalized cell lines, it is often difficult in nonimmortalized cell lines and primary cells due to low transfection efficiency. The use of siRNA technology in 3D organoid cultures has been very limited [48,49,50], likely also due to the technical challenge of delivering siRNAs through the extracellular matrix to organoids in situ [49]. Moreover, siRNA-mediated gene knockdown in organoid-derived monolayers has not been reported. In the current study, we successfully used siRNA technology in human jejunal organoid monolayers to individually silence expression of the two primary apical Na transporters in enterocytes. This allowed us to demonstrate, for the first time, that NHE3 and SGLT1 reciprocally regulate each other in normal human enterocytes.

Intestinal electrolyte transporters are known to influence one another to regulate absorptive and secretory processes. For example, coupled NaCl absorption is achieved by the dual operation of Na-H and Cl-HCO_3_ exchangers in the BBM of villus cells, and their regulation is mediated by intracellular pH [51,52]. In addition, several Na-dependent nutrient co-transporters including SGLT1 depend on the pump activity of the Na-K-ATPase at the basolateral membrane to maintain low levels of intracellular Na in order to provide a favorable electrochemical gradient necessary for their functioning [53,54]. Reciprocal regulation between NHE3 and SGLT1 was previously demonstrated in a rat intestinal epithelial cell line (IEC-18) [27]. Furthermore, intracellular cNO was shown to differentially regulate NHE3 and SGLT1 in vitro in IEC-18 cells [23,24,25] and in vivo in rabbit small intestine [25,55,56], which implies functional compensation between the two primary Na absorptive pathways. Molecular mechanisms for regulating BBM transporter function include changes in mRNA and total protein expression, changes in apical protein localization due to trafficking, and conformational changes that modulate transporter activity [21,57,58,59]. For the observed reciprocal regulation between NHE3 and SGLT1, changes could be mediated by various intracellular signaling pathways such as AMPK, cAMP/PKA, cGMP/PKG, and PKC [60,61,62,63,64,65]. Consistent with our previous studies, we recently demonstrated that cNO, which mediates its effects through cGMP/PKG signaling, also inhibits NHE3 but stimulates SGLT1 in human jejunal organoid monolayers, across multiple human specimens [18]. These results, together with the current study demonstrating that NHE3 and SGLT1 directly regulate one another, indicate that the cNO-mediated compensatory regulation of NHE3 and SGLT1 seen in mammalian intestinal models likely occurs also in human enterocytes.

Reciprocal compensatory regulation between transporters is unique, and likely functions in the maintenance of intestinal Na homeostasis in physiological conditions. This important observation will facilitate a better understanding of multiple common human diseases. For example, hypertension results from altered Na homeostasis [66], which this study suggests is a function of both coupled NaCl absorption (via the dual operation of Na-H and Cl-HCO_3_ exchangers), and SGLT1. Likewise, diabetes is secondary to altered glucose homeostasis [67], and this study suggests a critical role for NHE3 in the regulation of SGLT1, which is the predominant intestinal glucose transporter. As previously noted, our laboratory has published evidence that the compensatory regulation between the two primary Na absorptive pathways may be lost in obesity, leading to enhanced absorption of both Na and glucose, thus contributing to the pathophysiology of two common complications of obesity: hypertension and diabetes [8].

## 5. Conclusions

Herein, we performed siRNA-mediated knockdown studies for the first time in patient-derived jejunal organoid monolayers. Our results demonstrate that the two primary apical Na transporters NHE3 and SGLT1 reciprocally regulate each other’s function in normal human enterocytes. This proof-of-concept study will open the door to future studies investigating gene function in human enterocytes in the context of various diseases originating in the small intestine, from inflammatory bowel diseases to obesity-associated hypertension and diabetes.

## Data Availability

For verifiable requests, data will be made available.

## Supplementary Materials

The following supporting information can be downloaded at: https://www.mdpi.com/article/10.3390/cells13191623/s1; Figure S1: Original Western blot images for Figure 2. Figure S2: Original Western blot images for Figure 4.

## References

[1] Fair K.L., Colquhoun J., Hannan N.R.F. (2018). Intestinal organoids for modelling intestinal development and disease. Philos. Trans. R. Soc. B Biol. Sci..

[2] Beumer J., Clevers H. (2020). Cell fate specification and differentiation in the adult mammalian intestine. Nat. Rev. Mol. Cell Biol..

[3] Posovszky C. (2016). Congenital intestinal diarrhoeal diseases: A diagnostic and therapeutic challenge. Best Pr. Res. Clin. Gastroenterol..

[4] van der Heide F. (2016). Acquired causes of intestinal malabsorption. Best Pr. Res. Clin. Gastroenterol..

[5] Binder H.J. (2009). Mechanisms of Diarrhea in Inflammatory Bowel Diseases. Ann. N. Y. Acad. Sci..

[6] Seidler U., Lenzen H., Cinar A., Tessema T., Bleich A., Riederer B. (2006). Molecular Mechanisms of Disturbed Electrolyte Transport in Intestinal Inflammation. Ann. N. Y. Acad. Sci..

[7] Anbazhagan A.N., Priyamvada S., Alrefai W.A., Dudeja P.K. (2018). Pathophysiology of IBD associated diarrhea. Tissue Barriers.

[8] Palaniappan B., Arthur S., Sundaram V.L., Butts M., Sundaram S., Mani K., Singh S., Nepal N., Sundaram U. (2019). Inhibition of intestinal villus cell Na/K-ATPase mediates altered glucose and NaCl absorption in obesity-associated diabetes and hypertension. FASEB J..

[9] Sato T., Stange D.E., Ferrante M., Vries R.G.J., Van Es J.H., Van Den Brink S., Van Houdt W.J., Pronk A., Van Gorp J., Siersema P.D. (2011). Long-term Expansion of Epithelial Organoids from Human Colon, Adenoma, Adenocarcinoma, and Barrett’s Epithelium. Gastroenterology.

[10] VanDussen K.L., Marinshaw J.M., Shaikh N., Miyoshi H., Moon C., Tarr P.I., Ciorba M.A., Stappenbeck T.S. (2015). Development of an enhanced human gastrointestinal epithelial culture system to facilitate patient-based assays. Gut.

[11] Ettayebi K., Crawford S.E., Murakami K., Broughman J.R., Karandikar U., Tenge V.R., Neill F.H., Blutt S.E., Zeng X.-L., Qu L. (2016). Replication of human noroviruses in stem cell–derived human enteroids. Science.

[12] Noel G., Baetz N.W., Staab J.F., Donowitz M., Kovbasnjuk O., Pasetti M.F., Zachos N.C. (2017). A primary human macrophage-enteroid co-culture model to investigate mucosal gut physiology and host-pathogen interactions. Sci. Rep..

[13] Cheng Y., Hall T.R., Xu X., Yung I., Souza D., Zheng J., Schiele F., Hoffmann M., Mbow M.L., Garnett J.P. (2021). Targeting uPA-uPAR interaction to improve intestinal epithelial barrier integrity in inflammatory bowel disease. EBioMedicine.

[14] Kozuka K., He Y., Koo-McCoy S., Kumaraswamy P., Nie B., Shaw K., Chan P., Leadbetter M., He L., Lewis J.G. (2017). Development and Characterization of a Human and Mouse Intestinal Epithelial Cell Monolayer Platform. Stem Cell Rep..

[15] Tse C.-M., Yin J., Singh V., Sarker R., Lin R., Verkman A.S., Turner J.R., Donowitz M. (2019). cAMP Stimulates SLC26A3 Activity in Human Colon by a CFTR-Dependent Mechanism That Does Not Require CFTR Activity. Cell. Mol. Gastroenterol. Hepatol..

[16] Yin J., Tse C.-M., Avula L.R., Singh V., Foulke-Abel J., de Jonge H.R., Donowitz M. (2018). Molecular Basis and Differentiation-Associated Alterations of Anion Secretion in Human Duodenal Enteroid Monolayers. Cell. Mol. Gastroenterol. Hepatol..

[17] Hasan N.M., Johnson K.F., Yin J., Baetz N.W., Fayad L., Sherman V., Blutt S.E., Estes M.K., Kumbhari V., Zachos N.C. (2020). Intestinal stem cell-derived enteroids from morbidly obese patients preserve obesity-related phenotypes: Elevated glucose absorption and gluconeogenesis. Mol. Metab..

[18] Haynes J., Palaniappan B., Tsopmegha E., Sundaram U. (2022). Regulation of nutrient and electrolyte absorption in human organoid-derived intestinal epithelial cell monolayers. Transl. Res..

[19] Kiela P.R., Ghishan F.K. (2016). Physiology of Intestinal Absorption and Secretion. Best Pr. Res. Clin. Gastroenterol..

[20] Nikolovska K., Seidler U.E., Stock C. (2022). The Role of Plasma Membrane Sodium/Hydrogen Exchangers in Gastrointestinal Functions: Proliferation and Differentiation, Fluid/Electrolyte Transport and Barrier Integrity. Front. Physiol..

[21] Koepsell H. (2020). Glucose transporters in the small intestine in health and disease. Pflügers Arch. Eur. J. Physiol..

[22] Lehmann A., Hornby P.J. (2016). Intestinal SGLT1 in metabolic health and disease. Am. J. Physiol. Liver Physiol..

[23] Coon S., Kekuda R., Saha P., Talukder J.R., Sundaram U. (2008). Constitutive nitric oxide differentially regulates Na-H and Na-glucose cotransport in intestinal epithelial cells. Am. J. Physiol. Liver Physiol..

[24] Palaniappan B., Sundaram U. (2018). Direct and specific inhibition of constitutive nitric oxide synthase uniquely regulates brush border membrane Na-absorptive pathways in intestinal epithelial cells. Nitric Oxide.

[25] Palaniappan B., Manoharan P., Arthur S., Singh S., Murughiyan U., Sundaram U. (2019). Stimulation of constitutive nitric oxide uniquely and compensatorily regulates intestinal epithelial cell brush border membrane Na absorption. Physiol. Rep..

[26] Shah V., Lyford G., Gores G., Farrugia G. (2004). Nitric oxide in gastrointestinal health and disease. Gastroenterology.

[27] Coon S., Kekuda R., Saha P., Sundaram U. (2011). Reciprocal regulation of the primary sodium absorptive pathways in rat intestinal epithelial cells. Am. J. Physiol. Physiol..

[28] Vrhovac I., Eror D.B., Klessen D., Burger C., Breljak D., Kraus O., Radović N., Jadrijević S., Aleksic I., Walles T. (2014). Localizations of Na+-d-glucose cotransporters SGLT1 and SGLT2 in human kidney and of SGLT1 in human small intestine, liver, lung, and heart. Pflügers Arch. Eur. J. Physiol..

[29] Beucken T.v.D., Koch E., Chu K., Rupaimoole R., Prickaerts P., Adriaens M., Voncken J.W., Harris A.L., Buffa F.M., Haider S. (2014). Hypoxia promotes stem cell phenotypes and poor prognosis through epigenetic regulation of DICER. Nat. Commun..

[30] Wielenga M.C., Colak S., Heijmans J., Jeude J.F.v.L.d., Rodermond H.M., Paton J.C., Paton A.W., Vermeulen L., Medema J.P., Brink G.R.v.D. (2015). ER-Stress-Induced Differentiation Sensitizes Colon Cancer Stem Cells to Chemotherapy. Cell Rep..

[31] Foulke-Abel J., In J., Yin J., Zachos N.C., Kovbasnjuk O., Estes M.K., de Jonge H., Donowitz M. (2016). Human Enteroids as a Model of Upper Small Intestinal Ion Transport Physiology and Pathophysiology. Gastroenterology.

[32] Lima-Fernandes E., Murison A., Medina T.d.S., Wang Y., Ma A., Leung C., Luciani G.M., Haynes J., Pollett A., Zeller C. (2019). Targeting bivalency de-represses Indian Hedgehog and inhibits self-renewal of colorectal cancer-initiating cells. Nat. Commun..

[33] Ghishan F.K., Kiela P.R. (2014). Epithelial Transport in Inflammatory Bowel Diseases. Inflamm. Bowel Dis..

[34] Kiela P.R., Ghishan F.K. (2009). Ion transport in the intestine. Curr. Opin. Gastroenterol..

[35] Langerholc T., Maragkoudakis P.A., Wollgast J., Gradisnik L., Cencic A. (2011). Novel and established intestinal cell line models—An indispensable tool in food science and nutrition. Trends Food Sci. Technol..

[36] Meng Q., Choudry H.A., Souba W.W., Karinch A.M., Huang J., Lin C., Vary T.C., Pan M. (2005). Regulation of Amino Acid Arginine Transport by Lipopolysaccharide and Nitric Oxide in Intestinal Epithelial IEC-6 Cells. J. Gastrointest. Surg..

[37] Rahman S., Ghiboub M., Donkers J.M., van de Steeg E., van Tol E.A.F., Hakvoort T.B.M., de Jonge W.J. (2021). The Progress of Intestinal Epithelial Models from Cell Lines to Gut-On-Chip. Int. J. Mol. Sci..

[38] Collington G.K., Booth I.W., Knutton S. (1998). Rapid modulation of electrolyte transport in Caco-2 cell monolayers by enteropathogenic *Escherichia coli* (EPEC) infection. Gut.

[39] Jochems P.G.M., Garssen J., Van Keulen A.M., Masereeuw R., Jeurink P.V. (2018). Evaluating Human Intestinal Cell Lines for Studying Dietary Protein Absorption. Nutrients.

[40] In J.G., Foulke-Abel J., Estes M.K., Zachos N.C., Kovbasnjuk O., Donowitz M. (2016). Human mini-guts: New insights into intestinal physiology and host–pathogen interactions. Nat. Rev. Gastroenterol. Hepatol..

[41] Faber S.C., Lahoti T.S., Taylor E.R., Lewis L., Sapiro J.M., Sales V.T., Dragan Y.P., Jeffy B.D. (2022). Current Therapeutic Landscape and Safety Roadmap for Targeting the Aryl Hydrocarbon Receptor in Inflammatory Gastrointestinal Indications. Cells.

[42] Yamaura Y., Chapron B.D., Wang Z., Himmelfarb J., Thummel K.E. (2015). Functional Comparison of Human Colonic Carcinoma Cell Lines and Primary Small Intestinal Epithelial Cells for Investigations of Intestinal Drug Permeability and First-Pass Metabolism. Drug Metab. Dispos..

[43] Günther C., Winner B., Neurath M.F., Stappenbeck T.S. (2022). Organoids in gastrointestinal diseases: From experimental models to clinical translation. Gut.

[44] In J., Foulke-Abel J., Zachos N.C., Hansen A.-M., Kaper J.B., Bernstein H.D., Halushka M., Blutt S., Estes M.K., Donowitz M. (2015). Enterohemorrhagic Escherichia coli Reduces Mucus and Intermicrovillar Bridges in Human Stem Cell-Derived Colonoids. Cell. Mol. Gastroenterol. Hepatol..

[45] Teriyapirom I., Batista-Rocha A.S., Koo B.-K. (2021). Genetic engineering in organoids. J. Mol. Med..

[46] Sander J.D., Joung J.K. (2014). CRISPR-Cas systems for editing, regulating and targeting genomes. Nat. Biotechnol..

[47] Giuliano C.J., Lin A., Girish V., Sheltzer J.M. (2019). Generating Single Cell–Derived Knockout Clones in Mammalian Cells with CRISPR/Cas9. Curr. Protoc. Mol. Biol..

[48] Hiratsuka K., Miyoshi T., Kroll K.T., Gupta N.R., Valerius M.T., Ferrante T., Yamashita M., Lewis J.A., Morizane R. (2022). Organoid-on-a-chip model of human ARPKD reveals mechanosensing pathomechanisms for drug discovery. Sci. Adv..

[49] Morgan R.G., Chambers A.C., Legge D.N., Coles S.J., Greenhough A., Williams A.C. (2018). Optimized delivery of siRNA into 3D tumor spheroid cultures in situ. Sci. Rep..

[50] Zhang Q., Pan Y., Yan R., Zeng B., Wang H., Zhang X., Li W., Wei H., Liu Z. (2015). Commensal bacteria direct selective cargo sorting to promote symbiosis. Nat. Immunol..

[51] Zachos N.C., Tse M., Donowitz M. (2005). Molecular Physiology of Intestinal N^+^/H^+^ Exchange. Annu. Rev. Physiol..

[52] Sundaram U., Knickelbein R.G., Dobbins J.W. (1991). pH regulation in ileum: Na(+)-H+ and Cl(-)-HCO3- exchange in isolated crypt and villus cells. Am. J. Physiol. Liver Physiol..

[53] Thwaites D.T., Anderson C.M.H. (2007). H^+^-coupled nutrient, micronutrient and drug transporters in the mammalian small intestine. Exp. Physiol..

[54] Wright E.M., Hirayama B.A., Loo D.F. (2007). Active sugar transport in health and disease. J. Intern. Med..

[55] Coon S., Kim J., Shao G., Sundaram U. (2005). Na-glucose and Na-neutral amino acid cotransport are uniquely regulated by constitutive nitric oxide in rabbit small intestinal villus cells. Am. J. Physiol. Liver Physiol..

[56] Coon S., Shao G., Wisel S., Vulaupalli R., Sundaram U., Kekuda R., Saha P., Talukder J.R. (2007). Mechanism of regulation of rabbit intestinal villus cell brush border membrane Na/H exchange by nitric oxide. Am. J. Physiol. Liver Physiol..

[57] Broer S., Fairweather S.J. (2018). Amino Acid Transport Across the Mammalian Intestine. Compr. Physiol..

[58] Priyamvada S., Saksena S., Alrefai W.A., Dudeja P.K., Said H.M. (2018). Chapter 57—Intestinal Anion Absorption. Physiology of the Gastrointestinal Tract.

[59] Kiela P.R., Ghishan F.K., Said H.M. (2018). Chapter 56—Na+/H+ Exchange in Mammalian Digestive Tract. Physiology of the Gastrointestinal Tract.

[60] Dengler F., Rackwitz R., Pfannkuche H., Gäbel G. (2017). Glucose transport across lagomorph jejunum epithelium is modulated by AMP-activated protein kinase under hypoxia. J. Appl. Physiol..

[61] Arthur S., Coon S., Kekuda R., Sundaram U. (2014). Regulation of sodium glucose co-transporter SGLT1 through altered glycosylation in the intestinal epithelial cells. Biochim. Biophys. Acta (BBA)—Biomembr..

[62] Wright E.M., Hirsch J.R., Loo D.D.F., Zampighi G.A. (1997). Regulation of Na+/Glucose Cotransporters. J. Exp. Biol..

[63] Han Y., Bagchi P., Yun C.C. (2024). Regulation of the intestinal Na^+^/H^+^ exchanger NHE3 by AMP-activated kinase is dependent on phosphorylation of NHE3 at S555 and S563. Am. J. Physiol. Physiol..

[64] Lessa L.M.A., Carraro-Lacroix L.R., Crajoinas R.O., Bezerra C.N., Dariolli R., Girardi A.C.C., Fonteles M.C., Malnic G. (2012). Mechanisms underlying the inhibitory effects of uroguanylin on NHE3 transport activity in renal proximal tubule. Am. J. Physiol. Physiol..

[65] Nwia S.M., Li X.C., Leite A.P.d.O., Hassan R., Zhuo J.L. (2022). The Na+/H+ Exchanger 3 in the Intestines and the Proximal Tubule of the Kidney: Localization, Physiological Function, and Key Roles in Angiotensin II-Induced Hypertension. Front. Physiol..

[66] Jaitovich A., Bertorello A.M. (2010). Salt, Na+,K+-ATPase and hypertension. Life Sci..

[67] Galicia-Garcia U., Benito-Vicente A., Jebari S., Larrea-Sebal A., Siddiqi H., Uribe K.B., Ostolaza H., Martín C. (2020). Pathophysiology of Type 2 Diabetes Mellitus. Int. J. Mol. Sci..

