# Supplementary material for: Regulation of Enterocyte Brush Border Membrane Primary Na-Absorptive Transporters in Human Intestinal Organoid-Derived Monolayers
Source: Cells. 2024 Sep 28;13(19):1623. doi: 10.3390/cells13191623 (PMC11482628; doi:10.3390/cells13191623)
Supplement: Supplementary file 1 [file cells-13-01623-s001.zip › cells-3201848-supplementary.pdf]

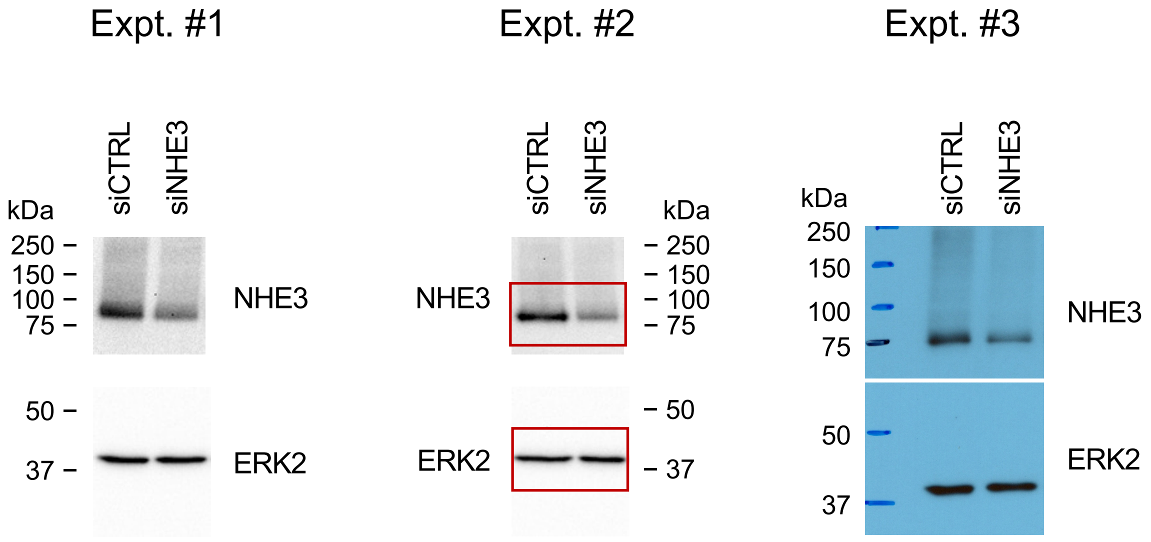

Figure S1. Original Western blot images for Figure 2. Membranes were cut horizontally into two pieces that were blotted with different primary antibodies (*Upper*: anti-NHE3; *Lower*: anti-ERK2). Boxed regions are shown in the main figure.

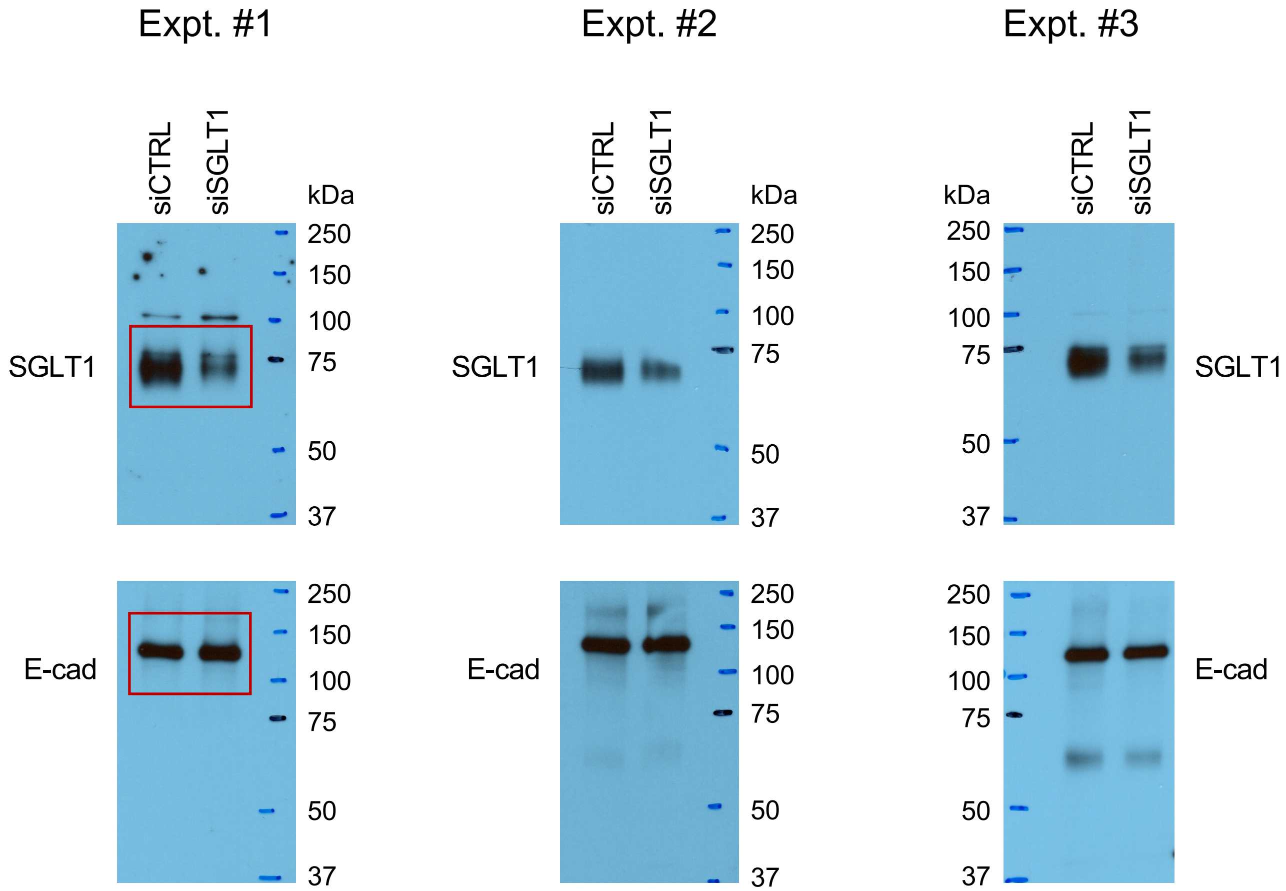

Figure S2. Original Western blot images for Figure 4. Membranes were first blotted with anti-SGLT1 primary antibody, then stripped and re-probed with anti-E-cadherin (E-cad) antibody. Boxed regions are shown in the main figure.
